# Radionuclide therapy: current status and prospects for internal dosimetry in individualized therapeutic planning

**DOI:** 10.6061/clinics/2019/e835

**Published:** 2019-07-22

**Authors:** Marcelo Tatit Sapienza, José Willegaignon

**Affiliations:** I Radiologia, Faculdade de Medicina FMUSP, Universidade de Sao Paulo, São Paulo, SP, BR; II Instituto do Cancer do Estado de Sao Paulo (ICESP), Hospital das Clínicas HCFMUSP, Faculdade de Medicina, Universidade de Sao Paulo, Sao Paulo, SP, BR

**Keywords:** Nuclear Medicine, Radionuclide Imaging, *In vivo* Dosimetry

## Abstract

The efficacy and toxicity of radionuclide therapy are believed to be directly related to the radiation doses received by target tissues; however, nuclear medicine therapy continues to be based primarily on the administration of empirical activities to patients and less frequently on the use of internal dosimetry for individual therapeutic planning. This review aimed to critically describe the techniques and clinical evidence of dosimetry as a tool for therapeutic planning and the main limitations to its implementation in clinical practice. The present article is a nonsystematic review of voxel-based dosimetry. Clinical evidence pointing to a correlation between the radiation dose and therapeutic response in various diseases, such as thyroid carcinoma, neuroendocrine tumors and prostate cancer, is reviewed. Its limitations include technical aspects related to image acquisition and processing and the lack of randomized clinical trials demonstrating the impact of dosimetry on patient therapy. A more widespread use of dosimetry in therapeutic planning involves the development of user-friendly dosimetric protocols and confirmation that dose estimation implies good efficacy and low treatment-related toxicity.

## INTRODUCTION

Radionuclide therapy was initially proposed in the mid-1940s and uses radioactive iodine isotopes to control hyperthyroidism or to destroy differentiated thyroid cancer cells 1. The use of iodine-131 for thyroid diseases still corresponds to the most frequent radionuclide therapy in nuclear medicine, although significant expansion of the use of new radiopharmaceuticals for diagnostic and therapeutic purposes in oncology is underway. The expansion of clinical indications has resulted from the development of new radiolabeled compounds as well as evidence of the response to therapy in randomized clinical trials, such as the use of somatostatin-lutetium-177 analogs for neuroendocrine tumors 2 and radio-223 for bone metastases of prostate carcinoma 3.

Although both nuclear medicine and radiotherapy use ionizing radiation for therapeutic purposes, it should be emphasized that radiopharmaceuticals administered in nuclear medicine are nonsealed sources, with wide distribution and metabolic interactions in the patient. Radiopharmaceuticals consist of a chemical compound (drug) combined with a radioactive isotope and exhibit extensive biological exchange in the organism due to their variable affinity for binding sites and excretion pathways. This pharmacokinetic behavior leads to the difficulty of accurately establishing the residence time of the radiopharmaceutical in each organ or tissue and makes it difficult to precisely determine the dose of radiation absorbed at the site. This is one of the main reasons that the planning of radionuclide therapy is usually based on the amount of radioactive substance (activity), expressed in Becquerel (1 Bq=1 disintegration per second) or Curie (1 Ci=3.7×10^10^ Bq), administered to the patient rather than the radiation absorbed dose, as measured by the energy deposited on a given mass of tissue, expressed in Gray units (1 Gy=1 joule/kg).

The efficacy and toxicity of radionuclide therapy are believed to be more directly related to radiation doses in target tissues and healthy tissues than to the administered radiopharmaceutical activity. However, despite recent advances in the field of internal dosimetry, with a good estimation of radiation doses, the method still has limited applications in therapeutic planning.

This review aimed to critically describe the techniques and clinical evidence of internal dosimetry as a therapeutic planning tool and the main limitations found in its implementation in clinical practice.

### Basics and methods of internal dosimetry

The radionuclides most commonly used in therapy emit particles with a low penetration range and high linear energy transfer (LET), leading to high ionization in the uptake site 4. Under some conditions, they also emit gamma radiation, which has high penetration and can be detected externally in the patient's body, allowing the imaging of the radiopharmaceutical biodistribution (Table 1). Radioisotope imaging obtained by planar scintigraphy, single-photon emission computed tomography (SPECT) and positron emission tomography (PET) scans is essential for determining the dose of radiation in a specific tumor or organ. Measurements of blood samples or whole-body detectors can also be used to estimate the dose of radiation in the bone marrow 5 but are less useful for the dosimetry of localized structures, such as tumors.

The image acquired for dosimetry purposes should be quantitative, going beyond simple image registration for the visual identification of normal and pathological structures. It is necessary to construct a map that reflects the actual activity in each structure or organ in real time, delimited by the regions of interest (ROIs) in planar imaging or the volumes of interest (VOIs) in SPECT or PET 6.

Quantitative data are more directly obtained in PET studies, in which the information is usually stored in units of activity per volume (Bq/mL), since the necessary corrections for quantification are incorporated into image acquisition and processing. However, a large number of studies in therapy describe the planar or SPECT imaging of gamma radiation, which requires several corrections to be adequately quantified 6,7. Among the required corrections, the following are highlighted:

#### Attenuation correction

Radiation from deep structures in the body is partially blocked / attenuated before reaching the detector. To retrieve this information, a correction factor is applied based on the X-ray (CT) available in PET/CT and SPECT/CT hybrid equipment. The CT provides a map of attenuation values for each voxel, expressed in Hounsfield units (HU).

#### Scatter correction

Radiation from an organ may not be blocked but is diverted from its initial trajectory by interacting with body tissues. The radiation, when diverted, loses part of its energy, and this energy difference makes it possible to make specific acquisitions to correct for the scattering, with the use of multiple energy windows.

#### Compensation of the collimator-detector response

events that lead to the loss of image quality upon interaction with the radiation detector, such as those arising from septal penetration and attenuation in the collimator, can be determined directly or by Monte Carlo simulation (point spread function). Its compensation during iterative image reconstruction allows resolution recovery in the SPECT images.

#### Calibration of the equipment

the acquisition of images from a phantom with known activity, including all the previously mentioned corrections, allows the relation between the number of counts detected in the image and the real activity to be established. This relationship can then be used to quantify patient images obtained with the same acquisition and processing parameters.

**Figure 1:**
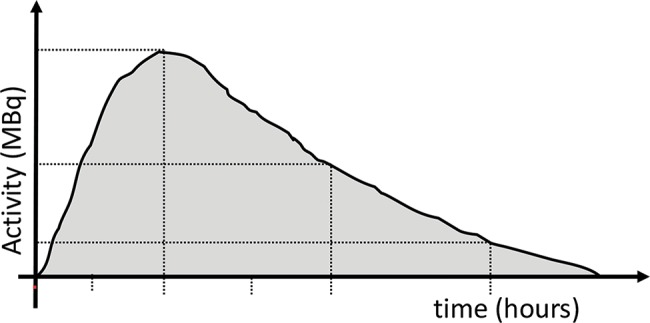
Time-activity curve: activity was quantified in consecutive images after drawing a region of interest (ROI) in the organ. The area under the curve corresponds to the accumulated activity (Ã), reflecting the total number of atoms that disintegrated in the region.

Dosimetry methods can be applied to estimate the doses of radiation received by the organs based on the detection and quantification of activity in a series of images acquired at different intervals after the administration of a radiopharmaceutical. The most widely used and validated method of internal dosimetry is the medical internal radiation dose (MIRD), idealized by the Internal Dosimetry Committee of the United States Society of Nuclear Medicine (SNM) and later adopted by the International Commission on Radiological Protection (ICRP) and the International Atomic Energy Agency (IAEA) 8. A graph of activity as a function of time can be constructed for a given organ. The area under the curve gives the total accumulated activity (Ã) in the organ over time (Figure 1). The division of Ã by the total amount of activity administered to the patient (Ao) results in the residence time of the radiopharmaceutical. These data, together with the patient characteristics (sex, age, and body weight), are applied in mathematical formulas to estimate the radiation absorbed dose per administered activity (Gy/Bq). The mathematical formalism employed by the MIRD can be summarized by the equation below: 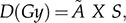


where D and Ã represent the radiation absorbed dose and accumulated activity in the organ, respectively, and S represents the dose constant that results from self-irradiation due to the accumulation of a specific radioisotope in a given organ added to the irradiation due to the accumulation of radioisotopes in other organs that affect the organ under study.

In addition to the residence time of the radiopharmaceutical in each organ, the S factor of the formula takes into account the physical characteristics of the radiation emitted by a specific radioisotope and the distance and geometric relation between organs. The geometry and its implications for interorgan irradiation are defined by the use of human body reference models. Thus, internal dosimetry using the MIRD formalism is based on the kinetics of accumulation and elimination of the radiopharmaceutical, which can be estimated through the quantification of images and geometric models of the human body. Although it is suitable for estimating the dose and population risk of probabilistic (stochastic) effects in which the absorbed radiation dose (Gray) is converted to equivalent and effective doses (expressed in Sieverts; 1 Sv=1 Gy for gamma or beta particles), the MIRD formalism is not always adequate for the estimation of the doses of radiation absorbed in a specific patient, since it does not consider individual variations in biodistribution and geometry.

In the context of the need for individualized dosimetry methods, the expansion of tomographic hybrid equipment – PET/CT and SPECT/CT – allowed the development of dosimetry methods, especially voxel-based dosimetry, based on real patient volumetric data 9,10. The concept of voxel dosimetry has already been incorporated into current MIRD publications for therapeutic planning 11,12. A voxel is the smallest element of a three-dimensional image, equivalent to a pixel in a planar image. Attenuation, scattering, resolution and calibration corrections are still necessary and even more critical for the quantification of a voxel to voxel activity. In addition, it is necessary to carefully adjust the positioning between the images acquired at different times so that a voxel of the same xyz coordinate corresponds to the same anatomical structure in consecutive images, and thus, the voxel cumulated activity (Ã) can be calculated.

**Figure 2:**
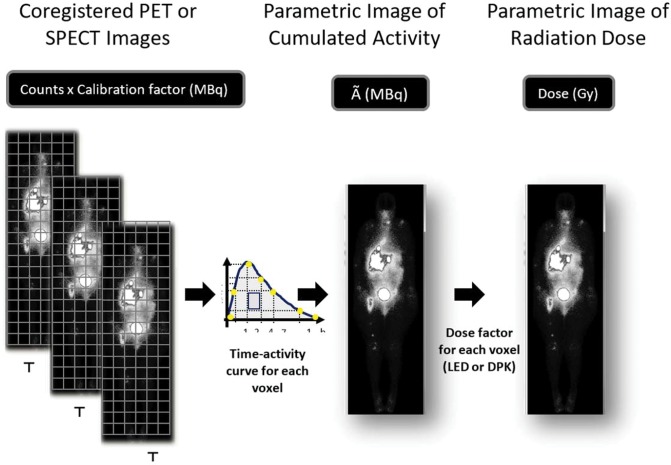
Sequential PET or SPECT images are coregistered so that a voxel of the same xyz coordinate always corresponds to the same structure. The calibration factor allows the transformation of counts in radioactivity (MBq). A parametric image of accumulated activity Ã (MBq.h) is obtained from the integral of activity through time in each voxel. The energy deposited in each voxel is calculated based on the local energy deposition (LED) or dose point kernel (DPK) and results in the radiation absorbed dose parametric image (Gy).

Voxel-based dosimetry follows a different sequence from the traditional method, since the initial delimitation of the organs is not necessary to quantify the cumulative activity (Ã) in a given organ. The first step is to adjust the spatial position of all sequential images so that a voxel of the same xyz coordinate always matches the same structure. The time-activity curve of each voxel xyz throughout the patient's body is then obtained, and the integration of these curves results in a tridimensional matrix that represents each voxeĺs Ã. The image representing the spatial distribution of the parameter Ã is used to estimate the radiation absorbed (Gy) in each voxel of the patient's body (Figure 2). Only at the end of this phase will the VOIs over the dose image be defined to estimate the radiation absorbed in a given organ or tumor.

The conversion from the parametric image “Ã” to the parametric image “Gy” is achieved by considering that the energy emitted by the radionuclide will either all be deposited in the voxel itself (local energy deposition – LED) or also be deposited in neighbor voxels with homogeneous attenuation (dose point kernel – DPK). Alternatively, Monte Carlo methods allow a more complete simulation of energy deposition, considering the random nature of particle interactions and tissue heterogeneity, despite the greater complexity and demand for resources and time 13. Both DPK and Monte Carlo simulations consider that, as in the conventional MIRD formalism, the radiation dose in a voxel will result from its self-irradiation added to the irradiation from neighboring voxels.

### Clinical evidence of internal dosimetry in radionuclide therapy planning

#### Differentiated thyroid carcinoma (DTC)

Radioiodine therapy is the most frequent therapy in nuclear medicine based on the expression of the sodium-iodine symporter (NIS) in differentiated thyroid carcinoma cells. Although radioiodine therapy is the most studied situation from the perspective of dosimetry, therapy is still usually performed based on the administered iodine-131 activity. Recent recommendations suggest the use of the administered activity of 30 to 100 mCi for thyroid remnant ablation, 100 to 150 mCi for adjuvant therapy, and 100 to 200 mCi for the treatment of a residual tumor or metastatic disease after surgery 14. Specifically, in the therapy of metastatic disease, there is a good response to the treatment of pulmonary micrometastases, with a low chance of a complete response in patients with macronodular pulmonary metastases or bone metastases. Among the main evidence is the comparison of survival in historical series 15 and retrospective studies that show a reduction in survival in patients with late indications of radioiodine therapy 16.

Dosimetric measures aimed at reducing treatment toxicity were proposed in the 1960s by restricting the bone marrow dose below 2 Gy and whole-body retention 48 h <120 mCi (<80 mCi in cases of diffuse lung uptake) 17. The administration of activities greater than or equal to 200 mCi results in a bone marrow dose >2 Gy in 11 to 22% of patients 18, which is the reason for limiting the treatment to 150 mCi in elderly patients (over 70 years old) for whom dosimetry is not performed 14. Although some studies show an increase in the complete response using a safe maximum dose compared to treatment with empirical activities in patients with metastatic or locally advanced disease 19, these data have not been confirmed in studies with a large series comparing the use of fixed 100 mCi activity (Gustave-Roussy – France) with the safe maximum dose (Memorial Sloan Kettering – USA) 20.

The main evidence of a relationship between tumor dosimetry and the response to complete decongestive therapy (CDT) with iodine-131 was obtained in Maxon's studies, in which a good probability of thyroid ablation was observed with doses >300 Gy and metastases with doses >85 Gy 21,22. A better response in lymph node metastases with doses >100 Gy was also reported in a concurrent study 23. A more recent study in which dosimetry was performed using PET with iodine-124 confirmed good responses in thyroid remnant ablation with doses above 300 Gy and 85 Gy in metastases, despite the limitations for dose determination in small volume lesions 24. A good correlation between the dose and remnant ablation has also been described with SPECT-based dosimetry 25. Although it is the first and most widely used modality for radionuclide therapy, there is a lack of randomized clinical trials of radioiodine therapy in CDT.

#### Neuroblastoma and malignant pheochromocytoma

Iodine-131 meta-iodobenzylguanidine (^131^I-MIBG) therapy is based on the uptake of adrenaline analogous to tumors of neuroectodermal origin that maintain amine reuptake mechanisms. Approximately 90% of neuroblastomas are avid and amenable to treatment with MIBG, with response rates above 30% in patients with refractory or recurrent neuroblastoma 26, as well as increasing indications for the induction or consolidation of therapy 27.

Usually, the therapy is prescribed empirically, with fixed or weight-adjusted activities (e.g., 200 mCi or 10 to 12 mCi/kg). The limiting toxicity may result from bone marrow irradiation, which usually ranges from 0.5 to 6 Gy, and bone marrow support or transplant is required in patients treated with high activities, which may reach 18 mCi or up to 50 mCi/kg 28. Two cycles of therapy, the first fixed (12 mCi/kg) and the second set by dosimetry to achieve a whole-body radiation dose of 4 Gy, is an option adopted by some centers with great success 27. Whole-body dosimetry is closely related to the bone marrow dose, and both are commonly used dosimetric parameters. Tumor dosimetry is rarely performed and is still considered a relatively complex procedure, requiring repeated images and measurements 29. Whole-body and bone marrow doses following ^131^I-MIBG therapy have been estimated, with reasonable safety and reproducibility 30, and in patients with neuroblastoma, the dose of whole-body radiation is correlated with the administered activity/kg; however, there is no clear correlation between the dose of radiation and the response to therapy or toxicity 28.

#### Neuroendocrine tumors

Somatostatin analogs labeled with beta particle emitter radionuclides, such as ^177^Lu-DOTATATE, are employed for the treatment of neuroendocrine tumors, which express somatostatin receptors in their cell membranes. The treatment is usually based on activity (4 cycles of 200 mCi), and its efficacy for progressing inoperable neuroendocrine tumors was demonstrated in a recent randomized clinical trial 2. The limiting toxicity is due to the radiation dose in the kidneys and bone marrow. Renal dosimetry may be used to adjust the administered activity in the last cycle of therapy. Although studies suggest a moderate correlation between dosimetry and the tumor response 31 and renal toxicity 32, there is still variability in the dose estimates according to the protocols of acquisition and image processing, which adds to the interpatient and radiopharmaceutical variation as well as variations in the dose estimate between cycles and between local or whole kidneys 33,34.

#### Yttrium-90 microsphere therapy for hepatic lesions

The efficacy of the radioembolization of hepatic lesions with ^90^Y particles has been demonstrated in clinical trials on primary liver tumors (hepatocarcinoma – HCC) 35 and metastatic tumors 36. Planar scintigraphy or SPECT performed after the intra-arterial injection of ^99m^Tc-MAA can be used to evaluate the biodistribution of the ^90^Y microspheres used in radioembolization, mainly in the definition of a pulmonary shunt or as an adjunct to angiography in the evaluation of a shunt to the digestive tract 37,38.

From a technical point of view, therapy with microspheres is possibly the simplest application of voxel-based dosimetry, since the radiopharmaceutical is retained in the tissue immediately after intra-arterial administration, without significant mechanisms of elimination other than physical decay 38. In this way, only one image is necessary, and there is no need to acquire and coregister a series of images. More frequently, voxel-based dosimetry is based on a SPECT/CT scan after the administration of a technetium-99m-labeled macroaggregate of albumin (^99m^Tc-MAA). Braking radiation (Bremsstrahlung) or the very limited positron emission of yttrium-90 can also be used for dosimetric studies after the procedure.

However, even with great technical simplicity, some factors of error or confusion should be noted 39, such as dose variation according to the tumor volume determination method (CT includes necrotic/hypoperfused areas and results in a greater volume and lower dose estimation compared to SPECT) and variation in the number and particle sizes of the MAA compared to ^90^Y-microspheres.

Retrospective studies have shown that an estimated dose >200-280 Gy in a primary or secondary liver tumor is associated with a high objective response, overall survival and disease-free survival 39,40. Although the estimated radiation dose is correlated with the tumor response and survival, the role of dosimetry in planning or enhancing therapy is still unclear. There is also no clear dosimetric determination of limits for irradiation to the liver or lung.

#### Radium-223 therapy for prostate carcinoma

Radium-223 (^223^Ra) is a radioisotope with high uptake in areas of bone remodeling, such as metastatic sites. The efficacy of ^223^Ra therapy was recently confirmed in a randomized clinical trial of patients with castration-resistant prostate cancer presenting with bone metastases and no visceral involvement 3. Therapy is based on a constant activity (50 kBq/kg) administered every 4 weeks for a total of 6 cycles. The radiation dose is approximately 10 times higher in the cortical bone than in the bone marrow, which justifies the low prevalence of bone marrow toxicity by the treatment 41,42. There is no clear correlation between the tumor radiation dose and the response to ^223^Ra therapy. Dosimetry is complicated in this scenario because ^223^Ra is an alpha particle emitter with high ionization in a very short trajectory. Therefore, precise knowledge of its location is essential for dose estimation. However, there is almost no emission of detectable gamma radiation, which makes it difficult to obtain images to determine the radionuclide distribution and kinetics.

### Limitations and challenges of clinical dosimetry

In addition to the methodological difficulties encountered in quantitative studies and the application of clinical dosimetry in the estimation of the dose of radiation received by the tissues, other limitations of technical and clinical nature contribute to the reduced number of dosimetric therapeutic plans.

#### Technical limitations

Several difficulties associated with the dosimetry method, including the need for careful correction of the attenuation, scattering and resolution of images, have been previously addressed. Even optimized images suffer from limited resolution, which leads to partial volume effects (spill-in and spill-out) and “blurring” of the structure outline. Image resolution can be defined as the ability of the system to distinguish and register two sources as distinct, and a limitation of this capability leads to imprecision in the positioning of the radiation source. If there is inaccuracy in the location of the radiopharmaceutical uptake area, the same imprecision will be present in the estimation of the radiation dose. For this reason, radiation dose estimation in lesions less than twice the resolution of the imaging system (i.e., approximately 2 cm in a SPECT study) should be considered underestimated 43. Additionally, the resolution of the anatomical image (CT) can be a limiting factor, and in situations where the volume or mass of the biological structure cannot be accurately measured (e.g., in pulmonary micrometastases, bone metastases, and subcentimetric lymph nodes), dose estimation may also be problematic, since the volume or mass that will receive the energy of the incident radiation is not accurately defined.

In addition to the uncertainty in the positioning of the radioactive event or the irradiated structure (due to the limited resolution of nuclear medicine and even of CT), other uncertainties present in PET or SPECT images will also be observed in dosimetry. For planning purposes, SPECT or PET should ideally be obtained before therapy during a diagnostic study. The small activities used in diagnostic studies imply low count rates and high statistical fluctuation in the number of counts per voxel and thus should be considered. This noise will propagate throughout all dosimetric procedures and may be erroneously assumed as a sign of dose heterogeneity. The use of dose histograms as markers of dose heterogeneity 44, implying small tumor responses (similar to the equivalent uniform dose – EUD – concept in radiotherapy), may be a misleading parameter in nuclear medicine. Additionally, the concept of the biological effective dose (BED) used in radiotherapy is less well defined in nuclear medicine due to the incomplete knowledge of biological responses in a low, continuous and decreasing dose rate.

Regarding reproducibility, it is difficult to state that the pharmacokinetic behavior observed in the therapy will be the same as that in the diagnostic study performed with “tracer” activities, since the physiological conditions of the organism can vary between two moments (e.g., TSH level in radioiodine therapy, renal function, and hydration) 45. In cases where internal dosimetry is performed to establish the dose resulting from the therapeutic cycle itself, with high activity and low noise in the images, dosimetric data are useful for the eventual planning of subsequent therapeutic cycles. Again, there is no guarantee that the pharmacokinetic behavior will be the same in each cycle, and it should be noted that tissues irradiated during the first cycle may undergo inflammatory changes or even cell death resulting from the treatment itself.

In addition to resolution, noise and reproducibility, it should be noted that among the sequential images combined to generate the parametric image of accumulated activity (Ã), there is a possible displacement of structures that would correspond to the same volume in the patient (e.g., intestinal loop in different locations within the abdomen). Overlap of the xyz coordinates of the corresponding voxels in the various images can be achieved by inelastic (without image deformity) or elastic (with image deformity) coregistration of the images.

#### Practical limitations

In addition to the abovementioned technical limitations, there are also practical limitations in the implementation of dosimetry-based therapy planning. Properly trained professionals in this field with mastery of both dosimetry and instrumentation concepts are rare. The training and hiring of these professionals would require investments from the nuclear medicine service, without clear economic returns. Outsourcing dosimetric procedures would require the integration of external professionals into the clinical routine through mandatory interfaces in quality control, scheduling, acquisition and image processing.

Hybrid imaging is essential for attenuation correction and consequently for image quantification used in voxel-based dosimetry. Although PET/CT is typically used for PET studies, the same does not apply to SPECT/CT, which is still scarcely available and associated with significantly high equipment costs. Multiple imaging time points needed for TAC integration also compete with examination slots needed in different clinical scenarios, such as baseline staging, the detection of relapse, treatment monitoring, increased costs and the impact of the procedure in a routine nuclear medicine service.

One possible minimization of these negative effects would be the use of simplified dosimetric estimates with fewer images (e.g., the use of only two SPECT images for dose estimation 33 or the association of a single SPECT image with other planar scans 6). This strategy allows the use of a single SPECT volumetric image with kinetic adjustment by data from previous studies 46. However, there should be a tradeoff between simplifying procedures and maintaining sufficient dosimetric accuracy to allow adequate therapy planning.

#### Radiobiological limitations

When internal dosimetry is performed using either the traditional organ-based method or the voxel-based method, the radiation absorbed dose is estimated in Gray units (1 Gy=1 joule/kg). However, the deposition of a certain amount of energy in an organ or a tissue is not the only factor related to a specific biological effect, such as cell death. The dose rate and dose distribution are important effect determinants in external beam radiotherapy, but their roles in internal dosimetry are not well understood.

Cells and tissues have the ability to repair radiation-induced damage, and this adaptive response should be considered in biological models. As time is required for a biological response to develop, the dose rates and fractionation have an impact on external radiotherapy effects. The concept of BED, which is based on the linear-quadratic model, allows the comparison of different dose rates that would lead to the same biological effect. However, even if the BED is a valid parameter for external beam radiotherapy, its value has not been clearly established in the ultralow dose rates of continuous radiation delivered from radionuclides 47.

The spatial distribution of the radiation dose in a target can also have a significant impact on its effects. One method used to assess dose heterogeneity in a tumor is the dose-volume histogram, which can be obtained in internal dosimetry methods based on voxels. The EUD establishes the equivalence of a heterogeneous dose with an evenly distributed dose in a tumor and could, in theory, be used as a parameter in the planning of radionuclide therapy. However, PET or SPECT images used in voxel-based dosimetry are a limited representation of the actual distribution of radioisotopes in the patient's body. The intrinsic statistical fluctuation leads to noise and therefore the introduction of heterogeneity in the images that does not correspond to the real distribution of the radionuclide in the patient. On the other hand, the low spatial resolution and partial volume effect lead to a loss of contrast and to homogenization of the dose estimate. There are no data available on how the heterogeneity of voxel-based dosimetry in radionuclide therapy should be interpreted.

The field of radiobiology also seeks a better understanding of the mechanisms related to bystander and abscopal effects 48 (effects of radiation on nearby and on distant nonirradiated cells, respectively), which together with different radiosensitivities of patients could lead to a nonlinear dose-response relationship in radionuclide therapy. Although it is increasingly important in the emergence of new clinical applications of radionuclide therapy, the correct interpretation of internal dosimetry parameters from a radiobiological point of view is not currently available. Reliable dosimetric protocols are a prerequisite to learn about the biological and medical interpretations of internal dosimetry in the research setting.

#### Clinical limitations

In the context of a nuclear medicine laboratory, internal dosimetry adds complexity and time to the daily routine, with the need to implement methods that are not fully standardized. Sequential multi-field-of-view SPECT scans are required for the quantification of cumulative activity and residence time for each organ or voxel, ideally in two stages in the accumulation phase and at least twice in the phase of elimination of the radiopharmaceutical in biological organs and tissues, not necessarily adding diagnostic information to the process 34.

Some oncological patients, especially those with advanced stage disease, often suffer from pain or limited breathing (e.g., due to ascites/pleura effusion) and may not tolerate long imaging exams. In regions with poor public transportation, traveling to the hospital to obtain delayed images can be challenging for patients in a reduced general condition. The whole dosimetric process, including processing and analysis, should be completed shortly after the last imaging to not delay the start of treatment, an issue that may be critical for fast growing tumors. It has not yet been clearly demonstrated that the benefits of individual dosimetry outweigh these negative effects, which could be reduced using a more straightforward approach with fewer images. The impact of a simplified dosimetric procedure in the final results regarding toxicity and tumor control has yet to be defined.

The incorporation of dosimetric procedures into the clinical routine would therefore need a strong foundation to support and justify its complexity and costs. However, as previously mentioned, radionuclide therapy has historically been established by empirical protocols based on the amount of activity administered to patients, and there is no clear demonstration of its benefits in terms of the tumor response or toxicity due to the implementation of dosimetric planning. Therefore, there is a vicious circle in which the implantation of dosimetry lacks justification based on clinical evidence, which in turn is difficult to obtain due to the low availability and adherence to clinical dosimetry protocols in nuclear medicine.

Based on the opinions of the authors, among the factors important for the clinical implementation of dosimetry, it would be desirable to have the following available:

- low-cost software for voxel-based dosimetry running in already existing work stations,
- a reduced number of images required – a minimum of one image at the maximum activity time (Tmax) associated with kinetic parameters or a delayed image (3 to 5x effective half-life),
- demonstration of the relation between the dose and measurable biological response, and
- trained personnel to perform internal dosimetry.

To contribute to these goals, our research group developed a dosimetric program at the Cancer Institute of the State of São Paulo (ICESP) in 2015/16 with the support of the Fundacao de Amparo a Pesquisa do Estado de São Paulo (FAPESP) in the Research Program for SUS (PPSUS process n° 2014/50091-5). The interactive software was developed to process SPECT/CT images in the MATLAB platform (Matrix Laboratory) with the following steps: the volumetric images were read and coregistered in Analyze format; the image matrix was converted to the activity matrix by voxels (MBq) through the application of conversion factors; the activity integral (by voxel) was calculated, resulting in a map of accumulated activity (Ã) by voxel; the map of Ã by DPK or LED factors was multiplied to obtain the radiation dose map by voxel (Gy); and the parametric dose images were reconverted to Digital Imaging and Communications in Medicine (DICOM) format, allowing further analysis in computer programs such as OsiriX (freeware). The developed code was renamed NMdose-VX, and the radiation dose provided by the code was compared to other dosimetry methods, such as thermoluminescent dosimetry (TLD) and OLINDA/EXM software, the latter being considered the standard method for internal dosimetry. NMdose-VX performed well in comparison with the indicated methods, allowing the use of patient images instead of idealized anthropomorphic models 49. Currently, the NMdose-VX code continues to be developed, with future prospects for making it more widely available.

## CONCLUSION

Voxel-based dosimetry allows internal dosimetry to be individualized, with the possibility of being incorporated into the therapeutic plan of patients. However, there are still technical and clinical limitations that hamper its broad use in the growing area of radionuclide therapy. Overcoming these limitations involves the development of user-friendly dosimetric protocols that are not costly and easy to implement in the clinical routine, along with the demonstration of its clinical benefits in well-conducted studies, which should clarify whether dosimetric therapy planning translates to decreased toxicity and good tumor responses.

## Figures and Tables

**Table 1:** Physical characteristics of commonly used radionuclides in therapy.

Radionuclide	Physical half-life (days)	Particle energy Beta (keV)	Maximal range (mm)
Iodine-131	8.0	610	2.0
Lutetium-177	6.7	496	1.6
Yttrium-90	2.7	2,290	11.9
